# Oxidative stress and reduced responsiveness of challenged circulating leukocytes following pulmonary instillation of metal-rich particulate matter in rats

**DOI:** 10.1186/s12989-014-0034-8

**Published:** 2014-08-15

**Authors:** Aaron Erdely, James M Antonini, Shih-Houng Young, Michael L Kashon, Ja K Gu, Tracy Hulderman, Rebecca Salmen, Terence Meighan, Jenny R Roberts, Patti C Zeidler-Erdely

**Affiliations:** 1 Health Effects Laboratory Division, National Institute for Occupational Safety and Health, Morgantown 26505, WV, USA; 2 Army Institute of Public Health, Aberdeen Proving Ground 21010, MD, USA; 3 NIOSH/HELD/PPRB, 1095 Willowdale Rd, MS-2015, Morgantown 26505-2888, WV, USA

**Keywords:** Microarray, Welding, Immunosuppression, Cardiovascular disease, Chromium, Whole blood cell gene expression

## Abstract

Welding fume is an exposure that consists of a mixture of metal-rich particulate matter with gases (ozone, carbon monoxide) and/or vapors (VOCs). Data suggests that welders are immune compromised. Given the inability of pulmonary leukocytes to properly respond to a secondary infection in animal models, the question arose whether the dysfunction persisted systemically. Our aim was to evaluate the circulating leukocyte population in terms of cellular activation, presence of oxidative stress, and functionality after a secondary challenge, following welding fume exposure. Rats were intratracheally instilled (ITI) with PBS or 2 mg of welding fume collected from a stainless steel weld. Rats were sacrificed 4 and 24 h post-exposure and whole blood was collected. Whole blood was used for cellular differential counts, RNA isolation with subsequent microarray and Ingenuity Pathway Analysis, and secondary stimulation with LPS utilizing TruCulture technology. In addition, mononuclear cells were isolated 24 h post-exposure to measure oxidative stress by flow cytometry and confocal microscopy. Welding fume exposure had rapid effects on the circulating leukocyte population as identified by relative mRNA expression changes. Instillation of welding fume reduced inflammatory protein production of circulating leukocytes when challenged with the secondary stimulus LPS. The effects were not related to transcription, but were observed in conjunction with oxidative stress. These findings support previous studies of an inadequate pulmonary immune response following a metal-rich exposure and extend those findings showing leukocyte dysfunction occurs systemically.

## Introduction

Pulmonary exposures to particulates, recognized for local effects in the lung, are now widely studied for their effects related to cardiovascular, immunological, and neurological dysfunction. Occupational exposure to welding fume is an exposure that is known to cause effects on extrapulmonary systems in all three categories. Welding joins two metals at high temperatures and generates an aerosol of gases and a fume that contains a metal-rich particulate fraction. Millions of workers weld as a part of their job duties with more than 430,000 full-time welders in the United States [1]. Data suggests that welders are immune compromised and in fact, welders are more prone to develop bronchitis and pneumonia [1] and it was recommended to vaccinate welders against pneumonia [2]. Animal studies support these findings and show localized immunosuppression by the inability of the lung to clear a bacterial infection following welding fume inhalation exposure [3]-[5]. Furthermore, exposure to manual metal arc stainless steel (MMA-SS) welding fume resulted in reduced responsiveness of lymphocytes from lung associated lymph nodes and the spleen [6].

Given the inability of pulmonary leukocytes to properly respond to a secondary stimulus the question arose whether dysfunction was also present in the circulating leukocyte population. Our aim was to evaluate the circulating leukocyte population in terms of cellular activation, presence of oxidative stress, and functionality after a secondary challenge, following welding fume exposure. Initially, gene expression changes in circulating leukocytes were analyzed. Previous studies by our laboratory have shown that circulating leukocytes rapidly respond to a pulmonary exposure with marked changes in relative mRNA levels [7]. Although informative, these data do not necessarily provide a functional endpoint. Therefore, circulating leukocytes were also challenged *ex vivo* to determine level of responsiveness by measuring stimulated protein production. In addition, relative gene expression changes were determined from mRNA isolated from challenged leukocytes to analyze differential effects. Lastly, the effect of welding particulate matter exposure to induce oxidative stress in circulating leukocytes was determined.

The results showed that metal-rich particulate matter pulmonary exposure has inhibitory effects on the circulating leukocyte population leading to a reduced responsiveness to a secondary stimulus. The effects were not related to transcription, but were observed in conjunction with oxidative stress. These findings support previous studies of an inadequate pulmonary immune response following a metal-rich exposure and extend those findings showing leukocyte dysfunction occurs systemically.

## Results

### Experimental design

**Figure 1:**
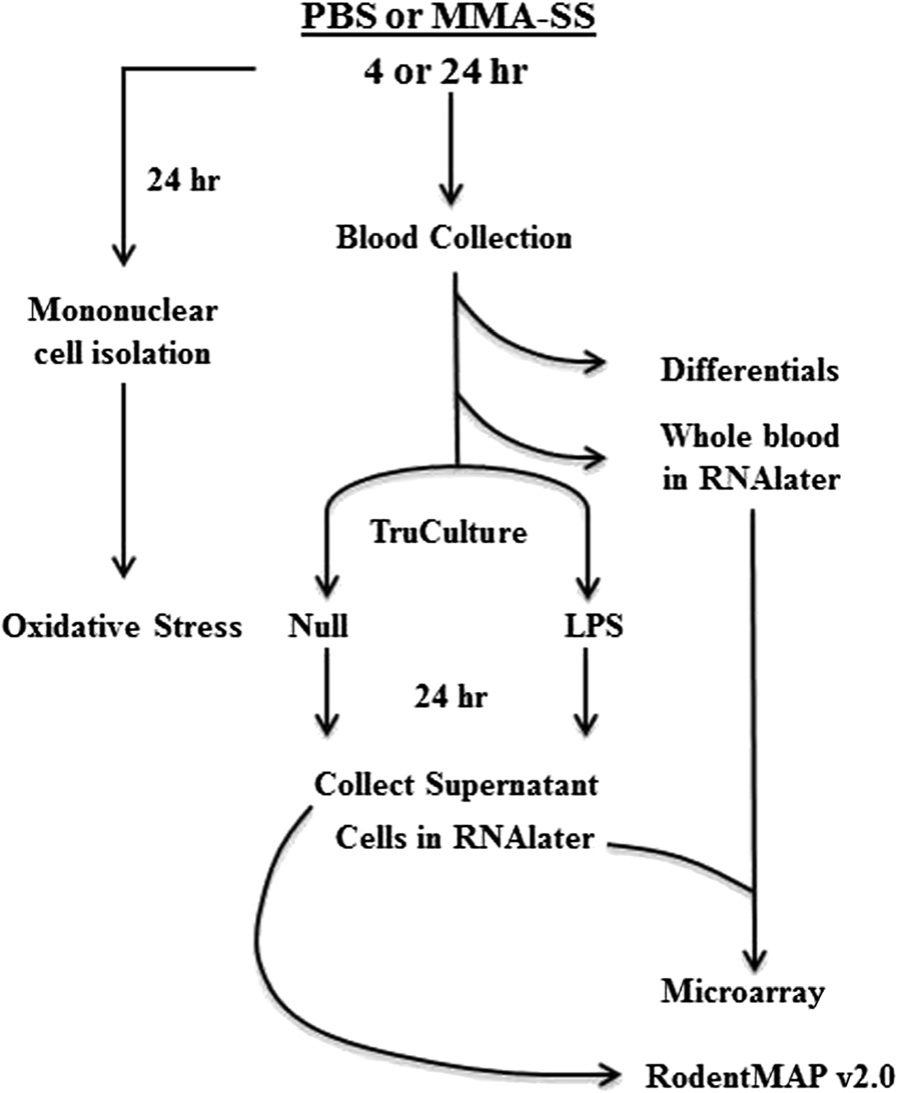
Experimental design of rats intratracheally instilled with either phosphate buffered saline (PBS) or manual metal arc stainless steel (MMA-SS) welding fume.

The complete experimental design is shown in Figure 1. Rats (n = 7 per group) were intratracheally instilled (ITI) with PBS or 2 mg of welding fume collected from a MMA-SS weld. Rats were sacrificed 4 and 24 h post-exposure by exsanguination under isoflurane anesthesia and whole blood was collected with heparin as the anticoagulant. Whole blood was used for cellular differential counts, RNA isolation with subsequent microarray and Ingenuity Pathway Analysis, and secondary stimulation utilizing TruCulture technology from Myriad/RBM as described in detail in the Materials and Methods section.

### Effect of MMA-SS welding fume exposure on total leukocytes and differentials

**Figure 2:**
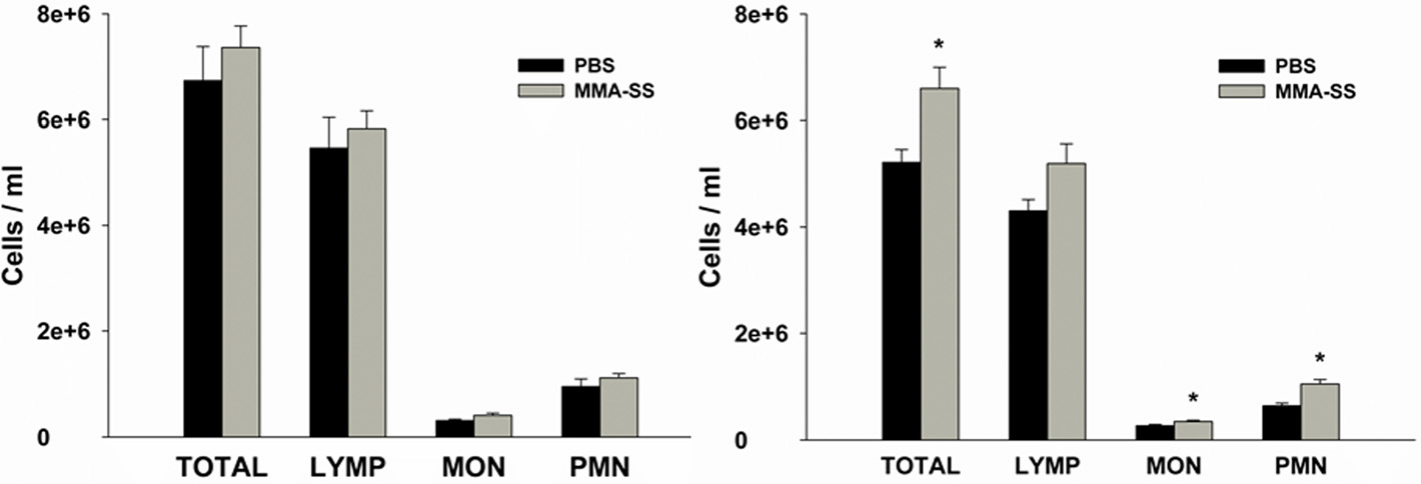
**Effect of MMA-SS welding fume instillation on circulating leukocytes.** The left panel shows changes 4 h post-instillation and the right panel shows changes at 24 h. *p < 0.05 vs PBS.

No significant changes in total leukocyte numbers or differentials 4 h after MMA-SS ITI (Figure 2, left panel) were measured. At 24 h after MMA-SS ITI (Figure 2, right panel) there was a statistical increase in total leukocytes with an increase in total monocytes and polymorphonuclear cells.

### Effect of MMA-SS ITI on circulating leukocytes gene expression

**Figure 3:**
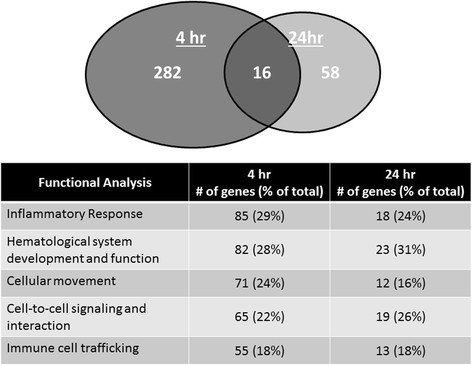
**Effect of MMA-SS welding fume instillation on relative mRNA expression in circulating leukocytes.** The Venn diagram represents genes altered due to MMA-SS welding fume exposure, only 16 genes overlapped between 4 and 24 h. The Table indicates the major functional categories altered and the number of genes in each.

Gene expression analysis showed significant changes at 4 h with 298 network eligible genes. At 24 h after ITI a 75% reduction in the number of eligible genes (74 in total) was found and only 5% of the genes (16 in total) altered at 4 h showed overlap (Figure 3). This indicated a rapidly evolving but controlled response. Cell-to-cell signaling and interaction, inflammatory response, immune cell trafficking, cellular movement, and hematological system development and function were the primary groups derived from the functional analysis (Figure 3). Interestingly, there was little difference in the functional categories despite the minimal overlap of similar genes further illustrating the rapid but controlled response (Figure 3).

Predicted activation or inhibition of upstream regulators based on the differential expression changes of the dataset at 4 and 24 h are shown in Table 1. Agreeing with a rapid but controlled response, significant changes were observed at 4 h with essentially a return to baseline by 24 h. Overall, the relative mRNA expression findings show significant cellular activation of circulating leukocytes with mechanisms related to inflammation and immune regulation.

**Table 1:** Predicted activation of molecules based on microarray analysis

**Molecule type**	**4 h**	**24 h**
Complex	PDGF BB; NFkB; IL12	
Cytokine	IFNG; IL6; TNF; TNFSF12; IL1B; TNFSF11; IL17A; IL13; IL2; Cxcl12; CSF2; C5; (IL1RN)	IL2
Enzyme	TGM2; CD44; PTGS2; CD38; NOS2; (TAB1)	TGM2
G-Protein coupled receptor	CCR2	
Group	Pkc(s); Ifnar; P38 MAPK; Ifn; Akt	
Growth factor	KITLG; FGF2	
Kinase	IKBKB; CHUK; PRKCD	
Transcription regulator	IRF7; CEBPA; STAT1; EGR1; STAT3; IRF1; NFKBIA; IRF5; CEBPB; IRF3; MTPN; (MYC); (TRIM24)	TP53
Transmembrane receptor	TLR3; TLR4; IFNAR1	

The identified molecules were derived from IPA upstream regulator analysis. All molecules had z-scores greater than 2.0 for a predicted activated upstream regulator and less than −2.0 for a predicted inhibited upstream regulator. Inhibited upstream regulators are shown in parentheses.

### Effect of MMA-SS welding fume exposure on LPS-induced protein levels

**Figure 4:**
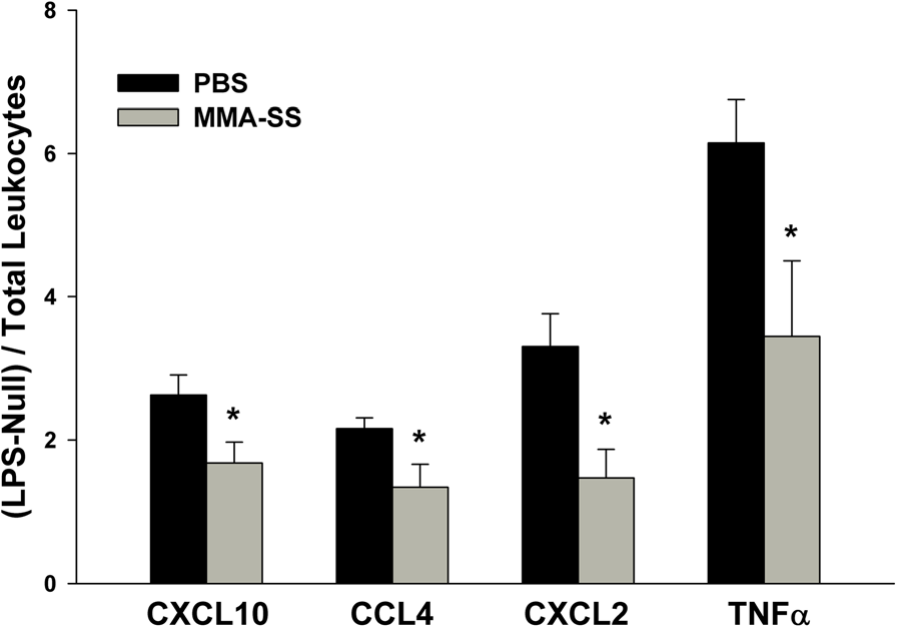
**Effect of MMA-SS welding fume instillation on protein production of challenged leukocytes.** Anticoagulated whole blood was collected 24 h post-instillation to PBS or MMA-SS and challenged with LPS (100 ng/ml). Supernatants were collected 24 h after LPS challenge and assayed for protein concentrations. Data are represented as (concentration)/(leukocytes/ml) times a factor of 10 to obtain a whole number. CXCL10 – pg/leukocyte* 100,000; CCL4 – pg/leukocyte* 10,000; CXCL2 – pg/leukocyte* 1,000,000; TNFα – ng/leukocyte* 100,000,000. *p < 0.05 vs PBS.

Whole blood was collected 4 and 24 h after MMA-SS or PBS ITI and incubated with or without LPS for 24 h. Cxcl10, Ccl4, Cxcl2, and TNF-α concentrations were determined from collected supernatants and data collected from the 24 h time point are shown in Figure 4. Data were generated by subtracting supernatant protein concentrations of null from LPS-challenged divided by the total leukocytes for each individual treated rat. It was necessary to divide by the total number of leukocytes because the TruCulture design is a specific volume of 1 mL irrespective of the number of circulating leukocytes. A significant decrease in LPS-induced protein production from circulating leukocytes harvested from rats exposed to MMA-SS fume for 24 h compared to PBS was found (Figure 4). However, despite a significant transcriptional response, there was no effect on LPS-induced protein production when comparing PBS and MMA-SS ITI stimulated whole blood collected 4 h post-exposure (data not shown).

### Effect of MMA-SS welding fume exposure on transcription following LPS challenge

**Figure 5:**
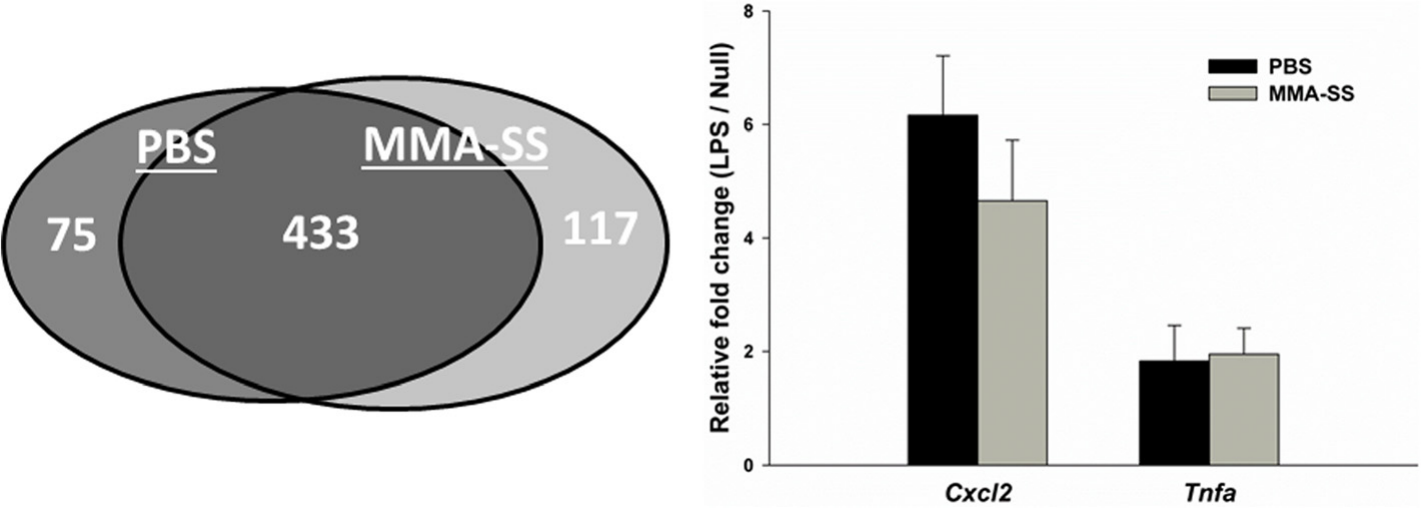
**Effect of LPS challenge on relative mRNA expression in whole blood cells.** Anticoagulated whole blood was collected 24 h post-instillation to PBS or MMA-SS and challenged with LPS (100 ng/ml). Whole blood cells were collected in RNA later 24 h after LPS challenge. The Venn diagram represents genes altered due to LPS challenge, 433 genes overlapped between PBS and MMA-SS treated rats. The figure shows no differential effect of LPS challenge after treatment for *Cxcl2* and *Tnfa*.

One potential mechanism for reduced protein production observed in whole blood cells collected 24 h post MMA-SS ITI then challenged with LPS is reduced transcription. Prior to secondary challenge with LPS, microarray indicated significant transcriptional activation of circulating leukocytes recovered from the welding fume group in reference to PBS shams. Analysis of gene expression from cells collected after secondary challenge to LPS showed induction of hundreds of differentially expressed genes (508 = PBS and 550 = MMA-SS; fold change 1.1, p < 0.05) with considerable overlap (433 genes) between the two exposures (Figure 5). Interestingly, interaction analysis, which compared the fold induction of LPS-induced genes from PBS compared to MMA-SS-exposed rats, showed only 8 genes were significantly altered. The data strongly indicated that LPS-induced transcriptional effects secondary to PBS and MMA-SS ITI were not affected by treatment despite the altered pre-LPS baseline at 4 h of relative mRNA expression of leukocytes from MMA-SS-exposed rats. In support, confirmatory gene expression by RT-qPCR showed no treatment effect for *Cxcl2* and *Tnfa* genes, two proteins significantly altered in Figure 4.

### Effect of MMA-SS welding fume exposure on oxidative stress in circulating leukocytes

**Figure 6:**
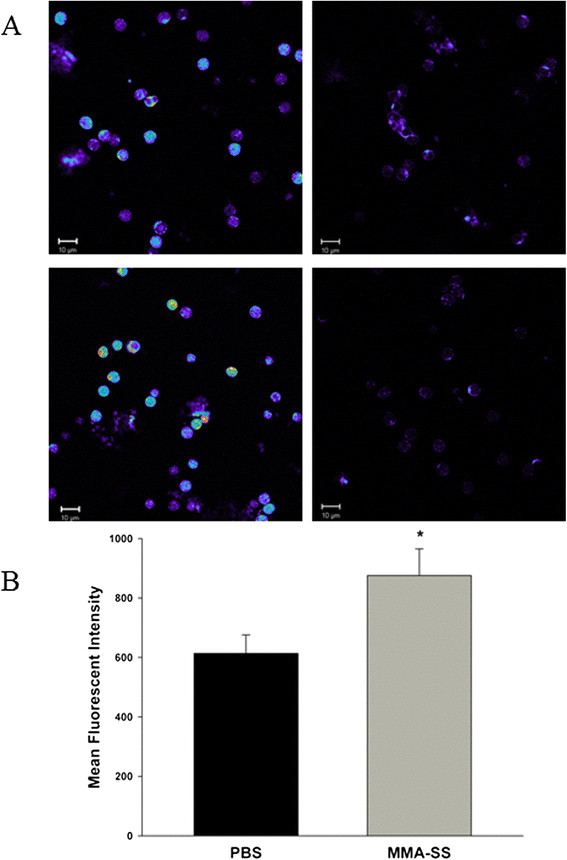
**Effect of MMA-SS welding fume instillation on mononuclear cell oxidative stress. (A)** Mononuclear cells were collected 24 h after instillation of PBS or MMA-SS, stained with H_2_DCFDA, fixed, and visualized by confocal microscopy for oxidative stress. Representative PBS exposed (upper left panel) and MMA-SS welding fume welding fume group (bottom left panel). Right panels represent background levels of unstained cells exposed to PBS (upper panel) and MMA-SS (lower panel). Fluorescent intensity was shown on a pseudo-color scale where the highest intensity staining is shown as white and red, followed by yellow, green, blue, and dark blue in order of decreasing intensity of the staining. The scale bar represents 10 μm. **(B)** Mean fluorescent intensity of isolated mononuclear cells incubated with H_2_DCFDA measured by flow cytometry. *p < 0.05.

Since reduced protein production was observed for cells collected at 24 h after treatment, oxidative stress was evaluated at that time point from isolated mononuclear cells. Representative images showed a greater level of oxidative stress in mononuclear cells isolated from the MMA-SS welding fume group (Figure 6A, bottom left panel) compared to PBS (Figure 6A, upper left panel). Background levels were similar between treatments (Figure 6A, right panels). For a quantitative evaluation of oxidative stress, isolated mononuclear cells incubated with H_2_DCFDA showed a significant increase in mean fluorescent intensity in the MMA-SS group compared to sham (Figure 6B). The increased oxidative stress complemented the qualitative confocal microscopy observations.

## Discussion and conclusion

The results from this study showed a reduced ability of circulating leukocytes to respond to a secondary stimulus after pulmonary instillation of MMA-SS welding fume in rats. The inability to produce inflammatory proteins in response to the secondary challenge was unrelated to transcription despite the altered expression profile observed prior to secondary stimulation. The reduced responsiveness of the circulating leukocytes was observed in conjunction with increased oxidative stress which provides insight into the contributing mechanism of the diminished response.

Increased oxidative stress in circulating mononuclear cells collected from welders has been shown [8]. Therefore, the current dosing regimen used in this study inducing systemic oxidative stress mimicked a real-life scenario in an occupational setting. The evaluation of oxidative stress was of interest because of potential effects on protein translation and the lack of any significant effects on induced gene expression following a secondary LPS challenge. Similar to measurements in welders [8], H_2_DCFDA was used to evaluate increased systemic oxidative stress. It should be noted that the dye used, H_2_DCFDA, will also react with reactive oxygen species other than hydroxyl radicals [9]. It has been shown that induced oxidative stress by H_2_O_2_ results in decreased protein translation initiation by the measure of an accumulation of ribosomes in the 80S peak [10]. Mechanisms for the reduced translation included increased phosphorylation of eIF2a dependent on Gcn2 and decreased ribosomal transit time [10]. Other reactive oxygen species, similar to H_2_O_2_, can also reduce protein synthesis [11]. These findings imply that underlying oxidative stress and the potential subsequent effect on translation may be contributory to increased susceptibility of welders to an infectious agent.

Several animal studies have found that welding fume exposure can result in localized immunosuppression because of the inability of the lung to clear an infection of *L. monocytogenes* after exposure [3]-[5]. Researchers hypothesized that the metal-rich particulate fraction of the welding fume caused an ineffective leukocyte response [1]. The specific mechanisms related to leukocyte suppression are unresolved but certainly the different metal constituents (e.g. chromium) of welding fume play an important role [1]. Of note, chromium and metal-rich residual oil fly ash also result in localized immune suppression, which suggests similar mechanisms initiated by metals [12]-[14]. Indeed, epidemiological evidence shows that welders are more prone to develop bronchitis and pneumonia compared to the general population [1],[15],[16], and it was recommended that welders be vaccinated against pneumonia [2]. The present study showed that leukocyte suppression was not confined to the lung but measurable in the circulating population. Effects on systemic leukocytes suggest a compromised cellular state of cells migrating into the lung for host defense further contributing to a localized susceptibility to infection.

Several mechanisms may contribute to the cellular activation and oxidative stress of circulating leukocytes which eventually lead to a diminished response. Exposure to MMA-SS welding fume causes lung cytotoxicity, increased pro-inflammatory cytokine production, DNA damage, as well as increased pulmonary oxidative stress as measured by lipid peroxidation [17],[18]. It has been hypothesized that circulating leukocytes may become activated by the inflammatory gradient following passage through the lung [7]. Systemic inflammation is also a known consequence of MMA-SS fume exposure [19]. Circulating inflammatory mediators and the acute phase response may lead to direct signaling effects on circulating leukocytes. Another mechanism potentially leading to dysfunction of circulating leukocytes is the translocation of soluble metals out of the lung. For the MMA-SS fume, 87% of the soluble fraction is chromium, which was found increased in the liver and kidney within hours post-exposure although the percent deposited in those organs was a very small proportion of the total pulmonary dose (<1.0%) [19]. The immunosuppressive effects of Cr are well documented [13]. Alveolar macrophages harvested from rats exposed to Cr had a reduced response *ex vivo* when stimulated with LPS [14]. In reference to MMA-SS, treatment with the total fume or individual metal components before infection showed that soluble Cr, as opposed to the Ni or Fe, resulted in reduced bacterial clearance [20]. Chromium levels in plasma correlated with urinary malondialdehyde, a marker of lipid peroxidation, in MMA-SS welders. In addition, MMA-SS welders had a significant decrease in lymphocyte glutathione concentrations indicating increased oxidative stress [21]. In a metal-rich PM_2.5_ exposure in humans, soluble chromium concentrations were associated with oxidative DNA damage in lymphocytes [22]. Also, in a rodent model, intraperitoneal injection of potassium dichromate caused increased oxidative DNA damage in peripheral lymphocytes [23]. Taken together, these studies indicate that systemic oxidative stress may be the result of leukocyte passage through the lung, release of inflammatory mediators causing systemic inflammation, and/or release of soluble reactive metals.

The analysis of gene expression changes comparing 4 and 24 h showed a very rapid induction of the response that was significantly diminished by 24 h. This was not unlike previous pulmonary particle exposures from our laboratory [7],[24]. Given that intravenous endotoxin administration in humans showed increased changes in gene expression peaking at 6 h and returning to baseline by 24 h, our findings were not unexpected [25]. Interestingly, the significant number of changed genes was associated with the time point and not with a significant increase in leukocyte number. This indicates activation of transcription as opposed to changes in cellular differentials. The molecular analysis revealed significant involvement of inflammatory and immune signaling. The rapid induction of these signaling pathways illustrates the speed at which the pulmonary exposure is identified systemically. From this study, it was unclear how the specific activated signaling pathways prior to the secondary challenge impacted the result of a diminished response.

While not specifically addressed in this study, the findings have implications for cardiovascular dysfunction as well. Humans and rodent studies have shown effects on heart rate variability, aortic augmentation index (a surrogate marker of arterial stiffness), markers of systemic inflammation and oxidative stress, and increased lesion area of atherosclerotic plaques after exposure to metal-rich welding particulate matter [8],[19],[26]-[28]. It has been shown that increased production of cellular reactive oxygen species directly contributed to vascular dysfunction following a pulmonary exposure to residual oil fly ash and the response appeared to be directly related to an increased rolling of leukocytes along the endothelium [29]. The rolling of leukocytes is mediated by adhesion molecules on the surface of endothelial cells. Studies from our laboratory show increased adhesion molecule expression in the vasculature from mice exposed to welding fume, as well as other particles. These studies, combined with the findings of systemic oxidative stress in welders and in this study, provide a contributing mechanism to explain cardiovascular dysfunction.

In conclusion, circulating leukocytes became compromised following a metal-rich particulate matter exposure. A probable factor in the leukocyte dysfunction was systemic oxidative stress, a situation that occurs in human welders. These findings illustrate the inability of circulating leukocytes to mount an adequate inflammatory response to a secondary stimulus and provide mechanistic insight as to why welders are more prone to infections.

## Materials and methods

### Study design and exposure

Male Sprague–Dawley [Hla:(SD) CVF] rats from Hilltop Lab Animals (Scottdale, PA, USA), weighing 250–300 g and free of viral pathogens, parasites, mycoplasmas, Helicobacter, and CAR Bacillus, were used for all exposures. The rats were acclimated for at least 6 days after arrival and were housed in ventilated polycarbonate cages on Diamond Dry cellulose chips and hardwood Sani-chips as bedding, and provided HEPA-filtered air, irradiated Teklad 2918 diet, and tap water *ad libitum*. The animal facilities are specific pathogen-free, environmentally controlled, and accredited by the Association for Assessment and Accreditation of Laboratory Animal Care International (AAALAC). All animal procedures used during the study were reviewed and approved by the National Institute for Occupational Safety and Health Animal Care and Use Committee.

The experimental design is shown in Figure 1. Rats (n = 7 per group) were intratracheally instilled (ITI) with PBS or 2 mg of welding fume collected from a MMA-SS weld. Rats were lightly anesthetized by an intraperitoneal injection of 0.6 ml of a 1% solution of sodium methohexital (Brevital, Eli Lilly, Indianapolis, IN, USA) then instilled with the welding fume sample suspended in 300 μl of sterile phosphate buffered saline (PBS). Sham controls were instilled with 300 μl of sterile PBS. Rats were sacrificed 4 and 24 h post-exposure by exsanguination under isoflurane anesthesia and whole blood was collected with heparin as the anticoagulant. Whole blood was used for cellular differential counts, RNA isolation with subsequent microarray and Ingenuity Pathway Analysis, and secondary stimulation utilizing TruCulture technology from Myraid/RBM as described below.

Assuming fume concentration (5 mg/m^3^, previous threshold limit value for welding fume), human minute ventilation volume (20,000 ml/min × 10^−6^ m^3^/ml), exposure duration (8 h/day × 60 min/h), and deposition efficiency (15%), it was calculated that the daily lung burden of a welder is approximately 7.2 mg. Using surface area of alveolar epithelium (rat = 0.4 m^2^; human = 102 m^2^) as a dose metric, the daily lung burden for a similar exposure in the rat is 0.0282 mg; 2 mg/0.0282 mg = 71 days of a worker exposed at 5 mg/m^3^ for 8 h/d.

### MMA-SS welding fume characterization

A bulk sample of welding fume was collected by Lincoln Electric Co. (Cleveland, OH). The fume was generated in a cubical open-front fume chamber (volume = 1 m^3^) by a skilled welder using manual metal arc welding with a flux-covered stainless steel electrode (MMA-SS; Murex 6011C Covered Electrode, Lincoln Electric Co., Cleveland, OH) and collected on 0.2 μm Nuclepore filters (Nuclepore Co., Pleasanton, CA) as previously described [18]. Particle size of the bulk sample of the collected fume was determined using scanning electron microscopy and found to be in the respirable size range with a count mean diameter of < 1.0 mm. The MMA-SS welding fume sample was stored in glass scintillation vials until ready for use. On the day of treatment, the fume sample was suspended in sterile phosphate-buffered saline (PBS), pH 7.4, and sonicated for 1 minute with a Sonifer 450 Cell Disruptor (Branson Ultrasonics, Danbury, CT). The total metal composition (weight%) measured by inductively coupled plasma atomic emission spectroscopy of the MMA-SS fume was Fe 41.1%, Mn 16.7%, Cr 28.5%, Ni 2.53%, Cu 0.40%, Ti 10.7%, V 0.11%. The soluble to insoluble ratio is 0.345. The weight% of metals in the soluble fraction are Fe 0.39%, Mn 11.7%, Cr 87.0%, Ni 0.65%, Cu 0.08%, Ti 0.13% [30].

### Leukocyte counts by flow cytometry

Flow cytometry on whole blood was performed as previously described [31]. Briefly, 100 μl of the blood cell suspension were added to a flow tube with 100 μl of 300 μg/ml mouse IgG (Sigma Chemical Co., St. Louis, MO, USA) in FACS buffer. After 10 minute incubation, 50 μl of pre-mixed antibodies in FACS buffer were added to the tube and stained for 30 minutes at room temperature on a shaker. The antibody mixture contained 5 μg/ml of the following antibodies: CD45R-FITC (clone HIS24), CD4-FITC (clone OX35), CD8-PerCP (clone OX-8), and CD3-APC (clone 1 F4). All the antibodies were purchased from PharMingen (Becton Dickinson, San Diego, CA, USA). Red blood cells were then lysed with 100 μl of Caltag Cal-lyse lying solution (GAS-010, Invitrogen, Carlsbad, CA, USA) for 10 minutes in the dark. Caltag counting beads (PCB-100, Invitrogen) were added for cell enumeration prior to analysis using a FACSCalibur flow cytometer (Becton Dickinson Biosystems, San Jose, CA, USA). Samples were acquired through a live gate without compensation. After collecting 3,500 counting beads, the data from all cells were exported to the analysis software, FlowJo (Treestar, Costa Mesa, CA, USA). Leukocytes were separated by side scattering (SSC) and forward scattering (FSC) into three gates: lymphocytes, monocytes, and eosinophils plus neutrophils. Total cell counts were verified by hemocytometer.

### Whole blood cell challenge

Whole blood was collected from rats under isoflurane anesthesia with heparin as an anticoagulant. Anticoagulated whole blood was inverted and immediately transferred to TruCulture tubes (Myriad/RBM). Since blood was not drawn directly into the TruCulture tubes, the transfer was done inside a sterile cell culture hood to minimize any potential contamination. After mixing, one mL of whole blood was transferred to a null (no stimulant) and a lipopolysaccharide (LPS) (100 ng/ml - stimulant) containing tube. Once added, the tubes were sealed, inverted to mix, and placed on a 37°C heat block. After 24 h, the cells had mostly settled. A plunger with a filtered tip was inserted to ensure separation of the cellular and supernatant fractions. The supernatant for each tube was collected and analyzed by Myriad/RBM for protein concentrations. Once the supernatant was collected, the plunger was removed and the cellular fraction was collected by adding 2.6 mL of RNAlater. RNA was isolated as described below.

### Oxidative stress measurement

Mononuclear cells were isolated using Histopaque in preloaded Accuspin tubes containing a frit (Sigma). Methodology was adapted from mononuclear cell responses from welders [8]. Cells were counted and resuspended in HBSS at 1 × 10^6^ per mL and incubated at 37°C for one hour. After the incubation, H_2_DCFDA at a final concentration of 10 μM was added to the cells and incubated at 37°C for 30 minutes. For flow cytometry, the monocyte population was gated using FSC vs SSC and the mean fluorescence intensity was quantitated for at least 10,000 gated cells. For confocal microscopy, the cells were then centrifuged and the pellet was washed in PBS followed by another centrifugation. The pellet was then suspended in 4% paraformaldehyde for 20 minutes. Cells were pelleted by centrifugation and the pellet was resuspended in 50 μl of Prolong Antifade (Invitrogen/Molecular probes). A 5 μl aliquot of cells was added to micro-well on a slide and cover-slipped. Cells were imaged with a laser scanning confocal microscope (Carl Zeiss, Inc., Thornwood, NY) at 63x with a 488 nm laser and an LP510 filter. Fluorescent intensity was shown on a pseudo-color scale where the highest intensity staining was shown as white and red, followed by yellow, green, blue, and dark blue in order of decreasing intensity of the staining.

### Gene expression

Whole blood RNA was isolated using the Mouse RiboPure™ Blood RNA Isolation Kit (Ambion, Austin, TX, USA) according to manufacturer’s directions and globin RNA was removed using the GLOBINclear™ kit (Ambion). A 2 μl aliquot of each RNA sample was quantified using a NanoDrop ND-1000 spectrophotometer (NanoDrop Technologies, Inc., Wilmington, DE, USA) and quality was assessed on the Agilent 2100 Bioanalyzer (Agilent Technologies, Palo Alto, CA, USA).

Labeled cRNA, from an input RNA of 375 ng, was prepared according to the manufacturer’s protocol, using the illumina TotalPrep RNA Amplification Kit (Ambion, Catalog #IL1791) for hybridization to the arrays. The labeled cRNA samples were then assessed for quality and quantity. To ensure consistency for the array hybridization, all cRNA samples for each time point were quantified at the same time. The illumina RatRef-12 beadchip contains 22,523 probes and allows twelve samples to be interrogated in parallel. After a 20 h hybridization period at 58°C, the beadchips were scanned using an illumina BeadStation 500G - BeadArray Reader (Illumina, Inc., San Diego, CA, USA). The microarray data were deposited to Gene Expression Omnibus (GEO) (http://www.ncbi.nlm.nih.gov/geo/) and are accessible through accession number (GSE58197). Confirmation of microarray results by quantitative real-time reverse transcription polymerase chain reaction (RT-qPCR) with hypoxanthine guanine phosphoribosyltransferase (*Hprt*) serving as the housekeeping gene was done as previously described [32].

### Statistics and data analysis strategy

Statistical analysis procedures for whole microarray datasets have been extensively described [32],[33]. Briefly, samples were imported into illumina® Beadstudio 3.0.19.0 and reference, hybridization control, stringency and negative control genes were checked for proper chip detection. Beadarray expression data were then exported with mean fluorescent intensity across like beads and bead variance estimates into flat files for subsequent analysis. Illumina BeadArray expression data were analyzed in Bioconductor using the ‘lumi’ and ‘limma’ packages. Gene lists containing group means of expression, p-values and standard fold changes were utilized as input for subsequent bioinformatics analysis.

The functional and upstream regulator analyses were generated through the use of Ingenuity Pathways Analysis (IPA) (Ingenuity® Systems, www.ingenuity.com). Whole datasets containing gene identifiers and corresponding expression values were uploaded into the application and a core analysis was done. Each identifier was mapped to its corresponding object in Ingenuity's Knowledge Base. The analysis criteria of fold change ±1.1 and p < 0.05, with PBS treated rats serving as the reference group, was derived from previous whole blood cell IPA analyses [32].

The Functional Analysis identified the biological functions and/or diseases that were most significant to the dataset. Molecules from the dataset that met the cutoff criteria defined above and were associated with biological functions and/or diseases in Ingenuity’s Knowledge Base were considered for the analysis. Right-tailed Fisher’s exact test was used to calculate a p-value determining the probability that each biological function and/or disease assigned to that data set is due to chance alone. The upstream regulator analysis utilizes the literature compiled in the Ingenuity knowledge base to derive causal effects between upstream regulators and targets. The analysis examines the known targets of each upstream regulator in the dataset, compares the targets actual direction of change to expectations derived from the literature then issues a prediction using a z-score algorithm. The direction of change is the gene expression in the experimental samples relative to a control. The analysis does not take into account the gene expression observed for the predicted upstream regulator itself because it may not differ between experimental and control samples. The analysis predicts the activity of the upstream regulator’s encoded protein.

For analysis other than microarray, all data are presented as means ± standard error. Since each time point contained respective controls and were harvested separately, all data were compared as sham versus exposed by Student’s *t*-test at each time point. Differences were considered statistically significant at p < 0.05.

## Competing interest

The authors declare that they have no competing interests.

## Authors’ contributions

AE conceived and designed the study and wrote the manuscript. JMA contributed to study design and treated all rats. SHY did the quantitative oxidative stress by flow cytometry. MLK and JKG conducted the statistical analysis. TH, RS, and TM aided in many technical aspects of the study. JRR did the confocal microscopy. PCZE performed microarray analysis in IPA. All authors read and approved the final manuscript.

## Authors’ information

The findings and conclusions in this report are those of the author(s) and do not necessarily represent the views of the National Institute for Occupational Safety and Health.
